# Association of erectile dysfunction and male lower urinary tract symptoms in a Japanese cross‐sectional survey

**DOI:** 10.1002/bco2.70219

**Published:** 2026-05-04

**Authors:** Katsuya Yamaguchi, Akihito Hashizume, Hiroki Ito, Yasushi Yumura, Shinnosuke Kuroda, Teppei Takeshima, Hisashi Hasumi, Daiki Ueno, Kazuhide Makiyama, Hiroji Uemura, Jun‐ichi Teranishi, Takashi Kawahara

**Affiliations:** ^1^ Departments of Urology and Renal Transplantation Yokohama City University Medical Center Yokohama Japan; ^2^ Department of Urology Yokohama City University School of Medicine Yokohama Japan; ^3^ Department of Reproductive Center Yokohama City University Medical Center Yokohama Japan

**Keywords:** ED, erectile dysfunction, IIEF‐5, lower urinary tract symptoms, LUTS

## Abstract

**Objective:**

The objective of this study is to evaluate the correlation between erectile dysfunction (ED) and lower urinary tract symptoms (LUTSs) using the International Index of Erectile Function‐5 (IIEF‐5) and the International Prostate Symptom Score (IPSS).

**Methods:**

An online survey was conducted among 4800 Japanese men using the International Index of Erectile Function‐5 (IIEF‐5), International Prostate Symptom Score (IPSS) and Overactive Bladder Symptom Score (OABSS). After excluding one incomplete response, data from 4799 participants were analysed. Participants were grouped by ED severity according to IIEF‐5 score. The total and subcategory scores of the IPSS and OABSS were compared among the groups. LUTSs were categorized as post‐micturition, storage, voiding symptoms and incontinence. An additional analysis was conducted by age group: younger (40s–50s) and older (≥70s).

**Results:**

Lower ED severity was associated with significantly lower IPSS and OABSS total scores (*p* < 0.001). Voiding symptoms and incontinence scores increased with ED severity (*p* < 0.001). Severe ED (IIEF‐5 to <8) was significantly associated with worse scores across all the LUTS domains. Younger men without ED had significantly lower scores in all LUTS categories (*p* < 0.001). However, among older men, no significant differences in LUTS scores were observed according to ED status.

**Conclusion:**

ED severity is positively correlated with LUTS severity, particularly voiding symptoms and incontinence. This relationship was more prominent in younger men.

## 1 INTRODUCTION

Erectile dysfunction (ED) and lower urinary tract symptoms (LUTSs) are common in older men.^1^, ^2^ With respect to the prevalence of LUTS, the EPIC study, conducted in 2005 in Canada, Germany, Italy, Sweden and the United Kingdom, involved 19 165 men and women.^3^ Of these, 7210 were male. The prevalence of urinary retention symptoms in men and women was 51.3% and 25.7%, respectively. Additionally, in men, the prevalence of all LUTS (urinary retention, urinary and post‐voiding symptoms) increased with age.

The prevalence of ED varies with age, country and region. According to Rosen et al.,^4^ the prevalence by age in Western countries is reported to be approximately 30.1% in their 50s, 51.1% in their 60s and 75.6% in their 70s. Nicolosi et al.^5^ surveyed 2400 individuals across four countries (Brazil, Italy, Malaysia and Japan). According to their study, age‐adjusted prevalence rates were 15.5% in Brazil, 17.2% in Italy, 22.4% in Malaysia and 34.5% in Japan.

Reports have indicated a close relationship between ED and LUTS. Rosen et al.^4^ evaluated the association between LUTS and ED in 12 185 individuals using the IPSS, Danish Prostatic Symptom Score (DAN‐PSS‐Sex) and IIEF. The results demonstrated that ED was strongly associated with the severity of LUTS in all age groups and in all groups with comorbidities (diabetes, hypertension, heart disease and hyperlipidemia) (*p* < 0.001). Additionally, Choi et al.^6^ reported an association between ED and LUTS in 1467 men of 20–60 years of age in Korea using the IIEF‐5 and IPSS. In this study, IIEF‐5 scores of 17–21 were defined as mild ED, and IIEF scores <17 were defined as moderate‐to‐severe ED for evaluation purposes. The results demonstrated that IIEF‐5 and IPSS scores were significantly negatively correlated (*r* = −0.243, *p* < 0.001). Additionally, among the seven IPSS items, Item 5 (urinary force) was reported to have the strongest correlation with the IIEF‐5 score (*r* = −0.243, *p* < 0.001). Furthermore, Item 3 (intermittency) and Item 7 (nocturia) of the IPSS were reported to be significantly associated with both mild ED and mild‐to‐moderate ED (OR 1.238, 95% CI 1.077–1.423, *p* = 0.003).

This study analysed survey results from 4799 Japanese men to examine whether ED correlates with LUTS and, specifically, which LUTS symptoms are associated with ED.

## 2 MATERIALS AND METHODS

A questionnaire‐based survey was administered using the internet. The questionnaire survey used a service provided by Ibridge (Freeasy, Tokyo, Japan). This service is a system where surveys we create are answered online by relevant survey monitors (men in their 40s to 60s) registered with iBridge Inc. iBridge Inc. has a large pool of approximately 13 million registered survey monitors, considered representative of the general Japanese population. Survey monitors receive a predetermined reward from iBridge for completing surveys.

This survey was collected between 20 August 2022 to 20 September 2022. Only adults who had been informed about the study and who agreed to participate in the study were included in the study. This study was approved by the institutional review board of Yokohama City University Medical Center (F240700009). Using this service, data were obtained from 4800 Japanese men. The respondents were divided according to age into 40s, 50s, 60s and ≥70s age groups, with 1200 questionnaires administered to each age group.

The International Index of Erectile Function‐5 (IIEF‐5) was used to diagnose and assess ED severity. The IIEF‐5 consists of five questions, with response options ranging from 1 to 5. The maximum and minimum scores were 25 and 5, respectively. Scores of ≥22 were considered normal; 17–21 points indicated mild ED, 12–16 points indicated mild‐to‐moderate ED, 8–11 points indicated moderate ED and 1–7 points indicated severe ED. In this study, the participants were evaluated and categorized into the following groups: normal (≥22 points), mild ED (17–21 points), mild‐to‐moderate ED (8–16 points) and severe ED (5–7 points).

IPSS is a scale used to assess the severity of LUTS associated with benign prostatic hyperplasia, select treatment options and evaluate treatment efficacy. The IPSS consists of seven questions with response options ranging from 0 to 5. The maximum and minimum scores were 35 and 0, respectively. Scores of 0–7 are classified as mild, 8–19 as moderate and 20–35 as severe. Additionally, the IPSS questions were categorized into post‐void symptoms (IPSS Item 1), storage symptoms (IPSS Items 2, 4 and 7) and voiding symptoms (IPSS Items 3, 5 and 6).

The Overactive Bladder Symptom Score (OABSS) is a symptom questionnaire specific to OAB. The OABSS consists of four questions, with the first question having options ranging from 0 to 2, the second ranging from 0 to 3 and the third and fourth ranging from 0 to 5. The maximum and minimum scores were 15 and 0, respectively. Scores of 0–5, 6–11 and 12–15 indicate mild, moderate and severe severity, respectively. The OABSS questions were as follows: Question 1 assessed daytime urinary frequency, Question 2 assessed nocturnal urinary frequency, Question 3 assessed urinary urgency and Question 4 assessed urinary incontinence.

In this study, 4800 participants completed questions from the IIEF‐5, IPSS and OABSS questionnaires, and the analysis was performed on 4799 people, excluding one person who did not answer the IIEF‐5 questions. IIEF‐5 scores were grouped according to severity (normal, mild, moderate and severe), and the total IPSS and OABSS scores for each group were compared and analysed.

Additionally, for each IIEF‐5 score severity group, we analysed the scores for each item in the IPSS related to post‐void symptoms (Item 1), urinary retention symptoms (Items 2, 4 and 7) and urinary symptoms (Items 2, 4 and 7). In the OABSS, we analysed the scores for the urinary incontinence item (Question 4) only, which was not included in the IPSS, for each IIEF‐5 severity group. In this study, we evaluated post‐void symptoms, storage symptoms and urinary incontinence in younger adults (40s–50s) and older adults (≥70s) according to the presence or absence of ED (Table 2). The presence or absence of ED was determined by classifying the IIEF‐5 score ≥22 group as the non‐ED group and the IIEF‐5 score ≤21 group as the ED group. Post‐void symptoms were evaluated using the IPSS 1 score; urinary retention symptoms using the IPSS Items 2, 4 and 7 scores; urinary symptoms using the IPSS 3, 5 and 6 scores; and urinary incontinence using the OABSS Item 4 score.

### 2.1 Statistical analysis

Age was analysed using the Mann–Whitney *U* test. The IPSS and OABSS scores for each IIEF‐5 severity level were evaluated using a one‐factor ANOVA. The tests were performed using GraphPad Prism (GraphPad Software, La Jolla, CA, United States). *p* values of <0.05 were considered to indicate statistical significance.

## 3 RESULTS

This study divided 4799 individuals into groups according to IIEF‐5 severity and analysed their IPSS and OABSS scores. Table 1 shows the patient background for this study (Tables 1 and S1). The numbers of patients per IEEF‐5 score group were as follows: ≥22 points, *n* = 974 (20.3%); 17–21, *n* = 837 (17.4%); IIEF‐5 score 8–16 points, *n* = 1910 (39.8%) and IIEF‐5 score <8 points, *n* = 1078 (22.5%).

**TABLE 1:** Characteristics of the study population.

	Number (%) median (mean ± SD)				
	IIEF ≧ 22	IIEF‐5 17–21	IIEF‐5 8–16	IIEF‐5 < 8	*p* value
Number of individuals	974 (20.3%)	837 (17.4%)	1910 (39.8%)	1078 (22.5%)	
Age (years)	53 (54.66 ± 10.16)	56 (57.10 ± 10.20)	59 (58.86 ± 10.94)	70 (67.04 ± 10.60)	<0.001
Numbers by age					
40s	372 (38.2%)	243 (29.0%)	488 (25.5%)	97 (9.0%)	<0.001
50s	298 (30.6%)	248 (29.6%)	505 (26.4%)	149 (13.8%)	<0.001
60s	205 (21.0%)	223 (26.6%)	496 (26.0%)	275 (25.5%)	<0.001
Over 70s	99 (10.2%)	123 (14.7%)	421 (22.0%)	557 (51.7%)	<0.001

**FIGURE 1:**
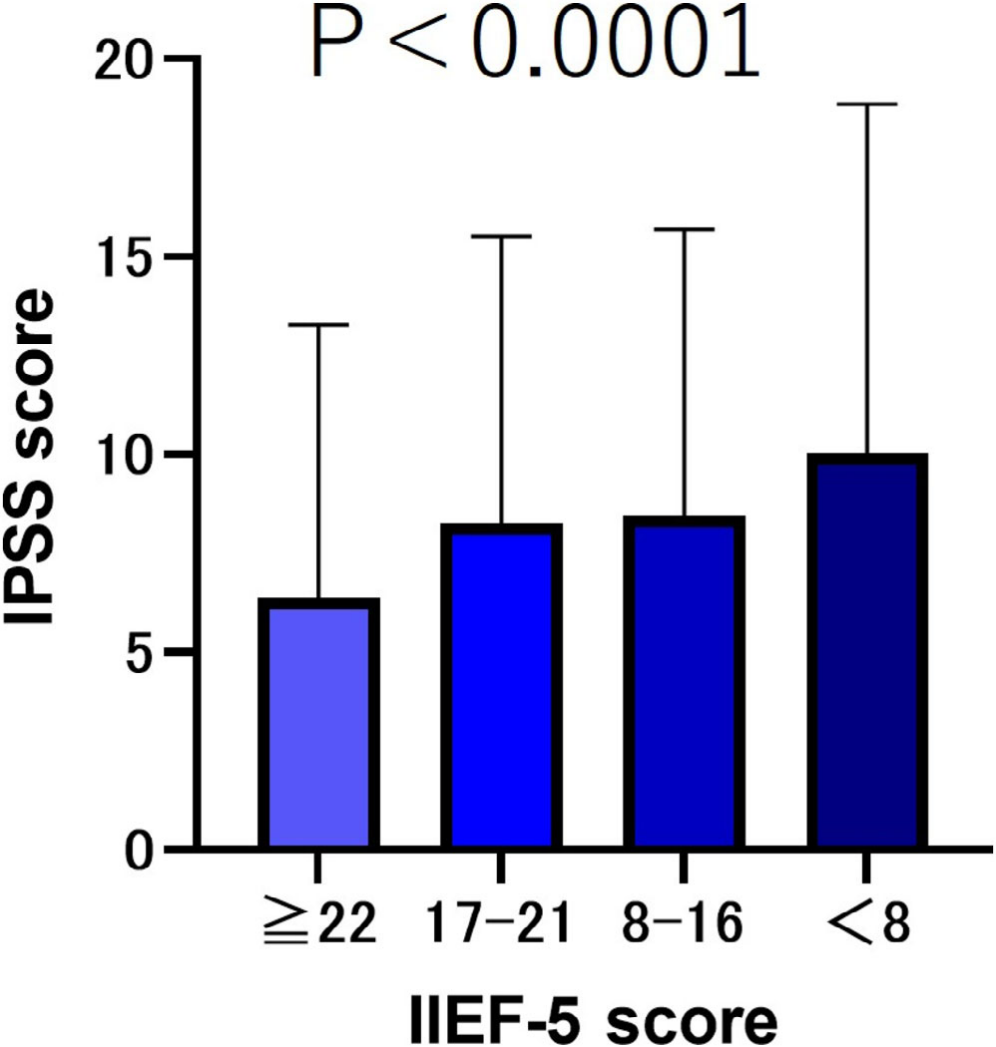
IPSS scores by severity of IIEF‐5. The total IPSS scores were as follows: IIEF ≥ 22 group, 4 (6.39 ± 6.90); IIEF‐5 score 17–21 group, 6 (8.27 ± 7.26); IIEF‐5 score 8–16 group, 6 (8.46 ± 7.23); and IIEF‐5 score <8 group, 7 (10.04 ± 8.78). The lower the severity of IIEF‐5, the lower the IPSS total score (*p* < 0.001).

**FIGURE 2:**
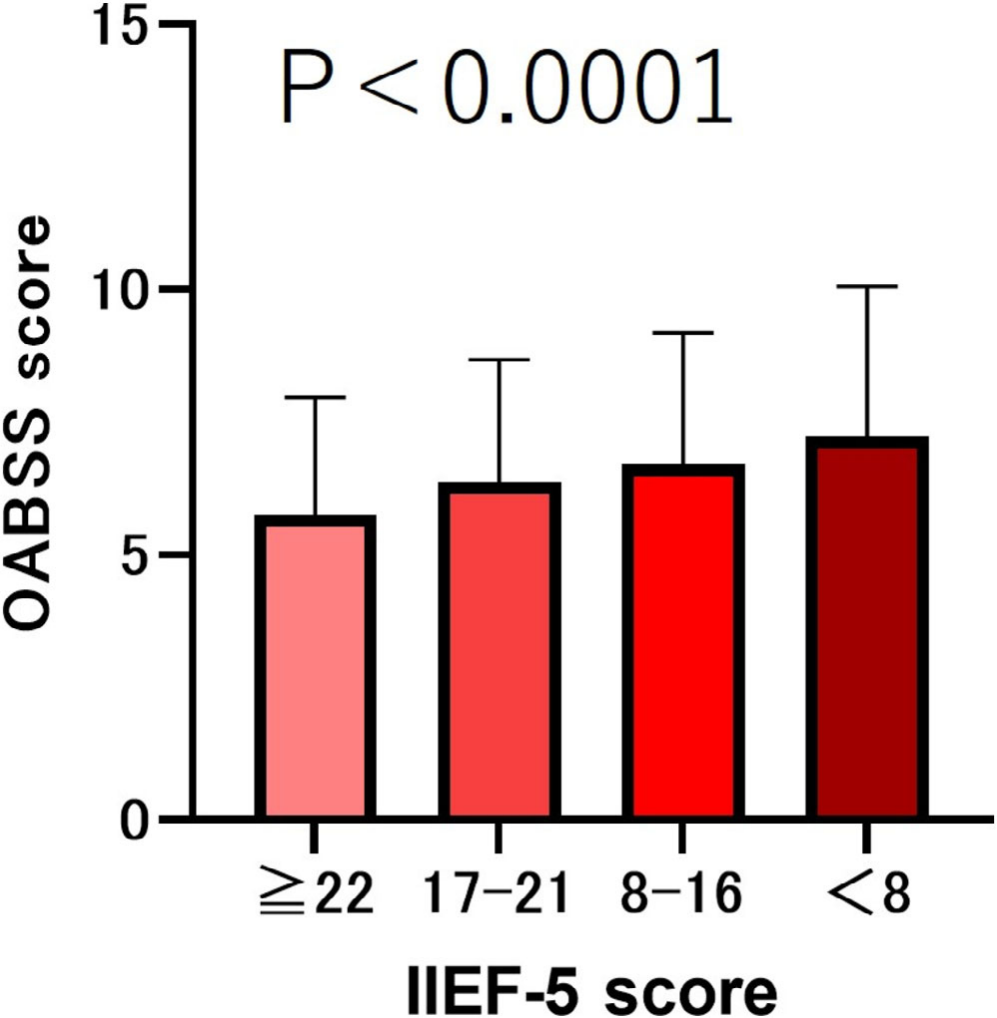
OABSS scores by severity of IIEF‐5. The total OABSS score was 5 (5.75 ± 2.21) in the IIEF‐5 ≥ 22 group, 6 (6.36 ± 2.33) in the IIEF‐5 score 17–21 group, 6 (6.71 ± 2.47) in the IIEF‐5 score 8–16 group and 7 (7.23 ± 2.81) in the IIEF‐5 score <8 group. The lower the severity of IIEF‐5, the lower the OABSS total score (*p* < 0.001).

The results showed that the older the age, the lower the IIEF‐5 score (*p* < 0.001). We compared the total IPSS scores based on the severity of the IIEF‐5 scores. The results showed that the lower the severity of IIEF‐5, the lower the IPSS total score (*p* < 0.001) (Figure 1). We compared the median (mean ± SD) OABSS total scores according to the severity of IIEF‐5 scores. The results showed that the lower the severity of IIEF‐5, the lower the OABSS total score (*p* < 0.001) (Figure 2).

**FIGURE 3:**
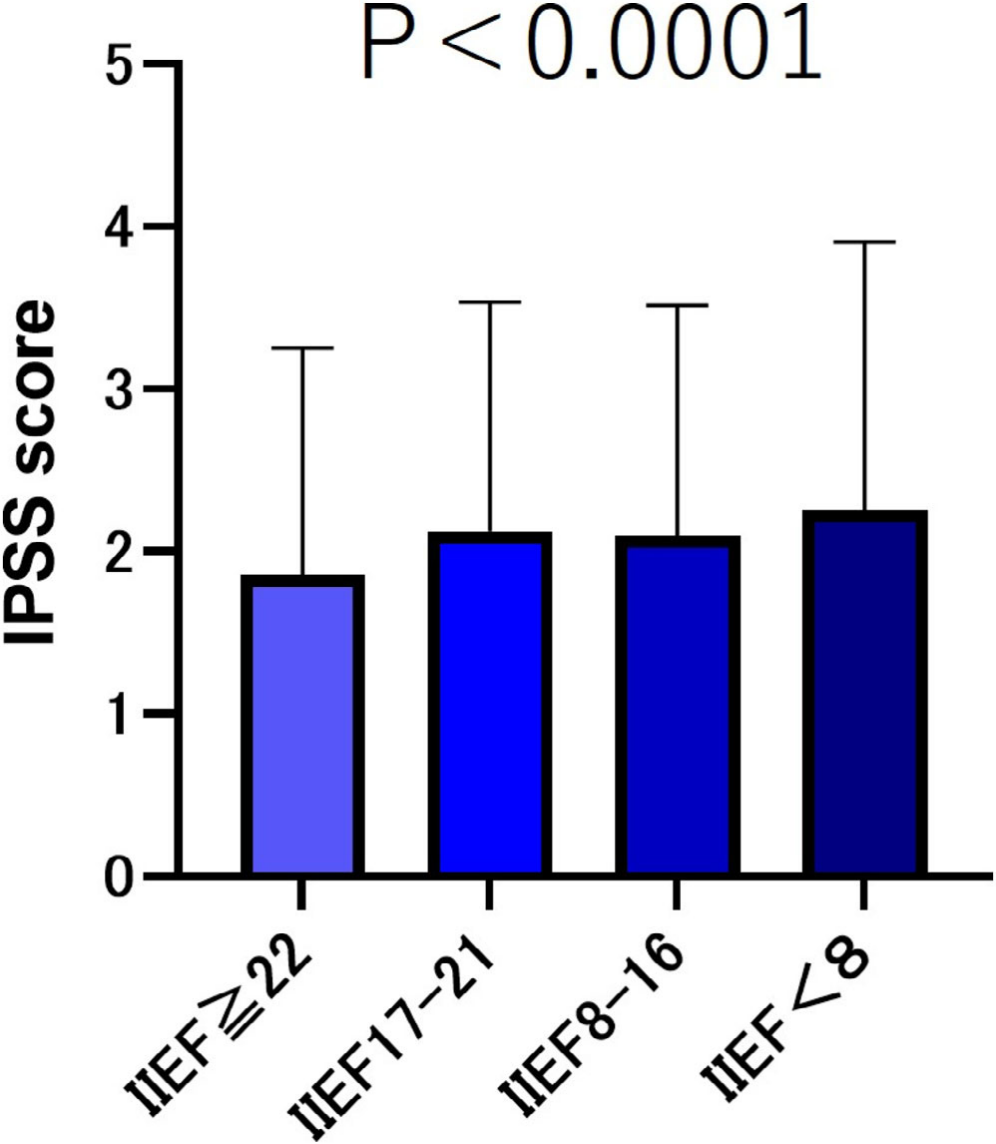
IPSS 1 (incomplete emptying) scores by severity of IIEF‐5. The IIEF‐5 score ≥22 group had a significantly lower IPSS 1 score than the IIEF‐5 score ≤21 group (*p* < 0.001). Additionally, there was no significant difference in the IPSS 1 scores of the IIEF‐5 score 17–21 group and IIEF‐5 score 8–16 group (*p* = 0.5752); however, both groups exhibited significantly lower IPSS 1 scores than the IIEF‐5 score <8 group (*p* < 0.001).

The IPSS 1 score (incomplete emptying) was compared based on the severity of IIEF‐5 scores. The results showed that the group without ED (IIEF‐5 score ≥22) had the lowest IPSS 1 score. The mild ED group (IIEF‐5 score 17–21) and moderate ED group (IIEF‐5 score 8–16) had similar scores, whereas the severe ED group (IIEF‐5 score <8) had the highest IPSS score (Figure 3).

**FIGURE 4:**
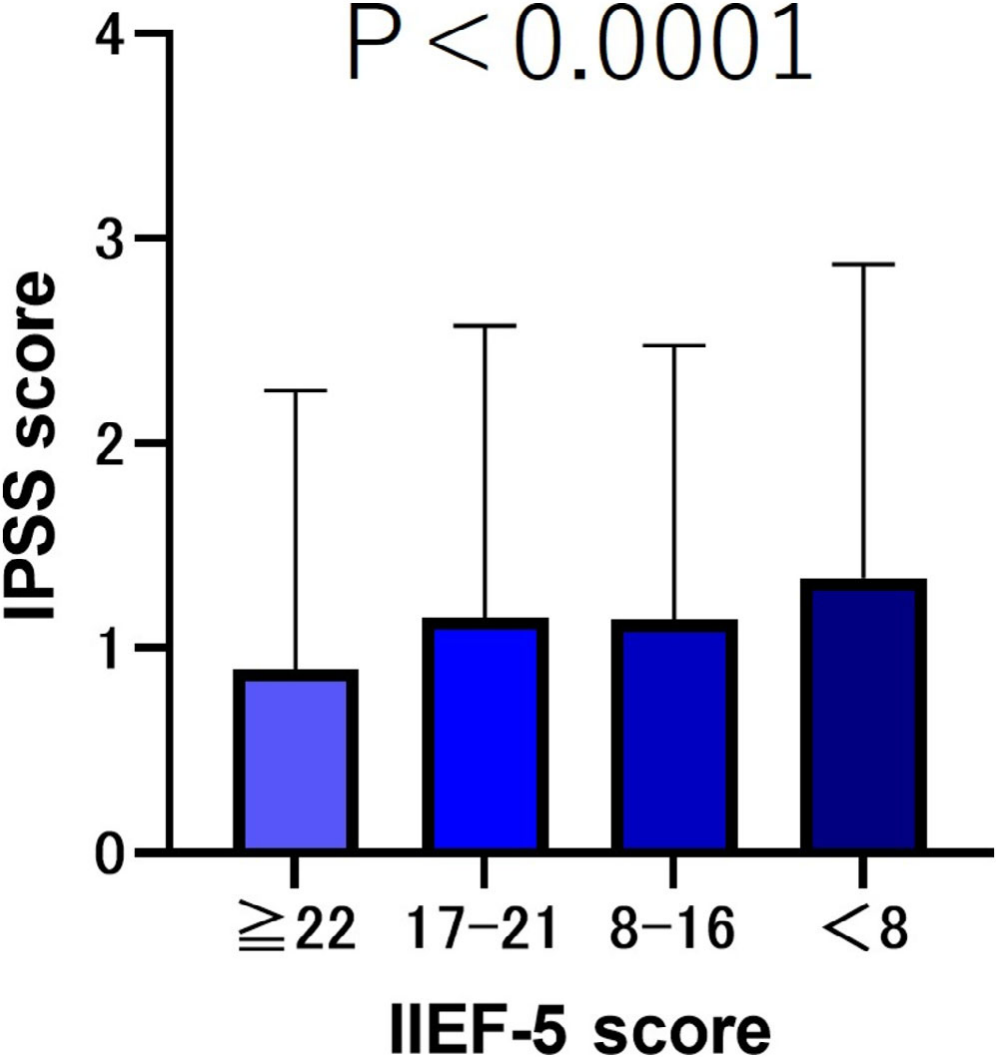
IPSS 2, 4 and 7 (storage symptoms) scores by severity of IIEF‐5. The IIEF‐5 score ≥22 group exhibited significantly lower total IPSS scores for Items 2, 4 and 7 than the IIEF‐5 score ≤21 group (*p* < 0.001). Additionally, there was no significant difference in the IPSS scores for Items 2, 4 and 7 between the IIEF‐5 score 17–21 group and the IIEF‐5 score 8–16 group (*p* = 0.294). However, both groups exhibited significantly lower IPSS scores than the IIEF‐5 score <8 group (*p* < 0.001).

We compared IPSS scores for Items 2, 4 and 7 (storage symptoms) according to the severity of the IIEF‐5 scores. The results showed that the group without ED (IIEF‐5 score ≥22) had the lowest IPSS 1 score. The mild ED group (IIEF‐5 score 17–21) and moderate ED group (IIEF‐5 score 8–16) had similar scores, whereas the severe ED group (IIEF‐5 score <8) had the highest IPSS score (Figure 4).

**FIGURE 5:**
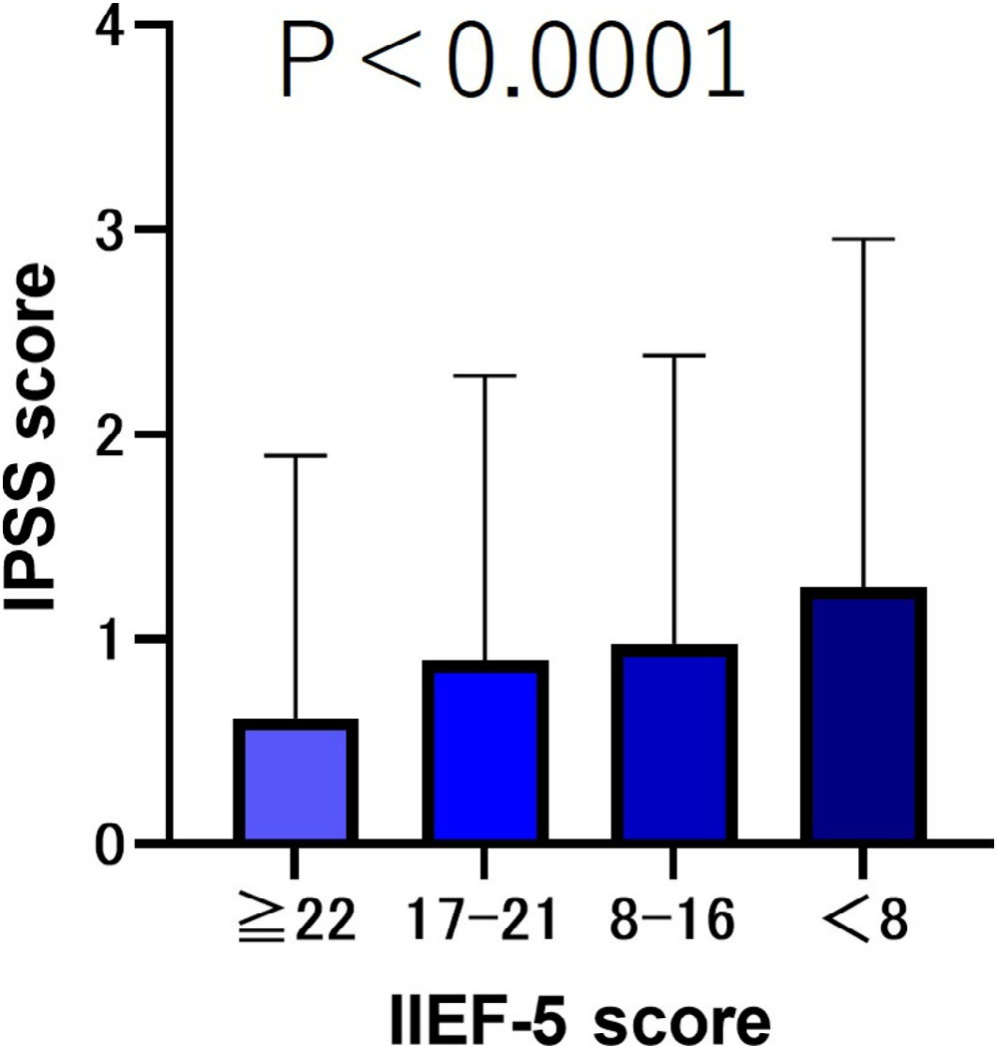
IPSS 3, 5 and 6 (voiding symptoms) scores by severity of IIEF‐5. The lower the severity of IIEF‐5, the lower the IPSS scores for Items 3, 5 and 7 (*p* < 0.001).

We compared IPSS scores for Items 3, 5 and 7 (voiding symptoms) according to the severity of the IIEF‐5 score. The results showed that the lower the severity of the IIEF‐5 score, the lower the IPSS scores for Items 3, 5 and 7 (*p* < 0.001) (Figure 5).

**FIGURE 6:**
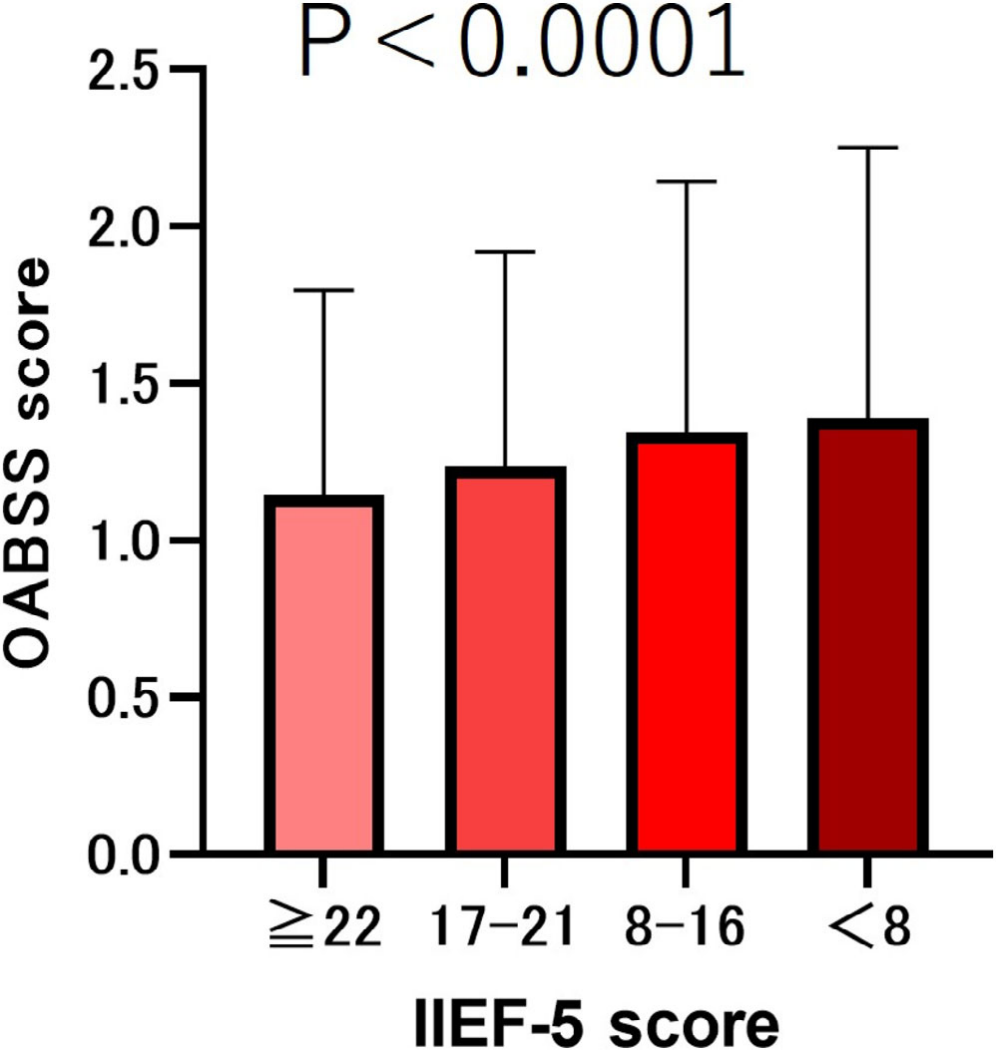
OABSS 4 (incontinence) scores by severity of IIEF‐5. The lower the severity of IIIEF‐5, the lower the score for OABSS Item 4 (*P* < 0.001).

A comparison of OABSS Item 4 scores (urinary incontinence) was conducted according to the severity of IIEF‐5 scores. The results showed that the lower the severity of IIEF‐5, the lower the OABSS Item 4 score (*p* < 0.001) (Figure 6).

The study population was divided into two groups: younger adults (40–50 years) and older adults (≥70 years). Post‐void symptoms, urinary retention symptoms and urinary incontinence were evaluated according to the presence or absence of ED (Table 2). The IIEF‐5 score ≥22 group was classified as having no ED, whereas the IIEF‐5 score ≤21 group was classified as having ED. In the younger age group, the ED group exhibited significantly lower scores than the non‐ED group in all categories, including the IPSS total score, post‐void symptoms, urinary retention symptoms, urinary symptoms and urinary incontinence (*p* < 0.001). In contrast, among the elderly population, although the score for urinary retention symptoms was lower in the group without ED compared to the group with ED (*p* = 0.0038), no significant differences were observed for the IPSS total score, post‐void symptoms, urinary symptoms or urinary incontinence.

**TABLE 2:** Relationship between IIEF‐5 and IPSS by age group.

	Median (mean ± SD)		
	IIEF‐5 ≧ 22	IIEF‐5 ≦ 21	*p* value
40–50s			
IPSS total score	4 (6.09 ± 6.71)	5 (7.66 ± 7.22)	<0.001
IPSS incomplete emptying (No. 1)	1 (1.90 ± 1.43)	2 (2.11 ± 1.46)	<0.001
IPSS stage score (No. 2, 4 and 7)	0 (0.86 ± 1.37)	1 (1.03 ± 1.37)	<0.001
IPSS voiding score (Nos. 3, 5 and 6)	0 (0.54 ± 1.23)	0 (0.82 ± 1.32)	<0.001
OABSS incontinence (No. 4)	1 (1.11 ± 0.59)	1 (1.30 ± 0.79)	<0.001
Over 70s			
IPSS total score	6 (9.13 ± 8.36)	8 (10.36 ± 8.23)	0.0565
IPSS incomplete emptying (No. 1)	1 (2.00 ± 1.52)	2 (2.18 ± 1.50)	0.1025
IPSS stage score (Nos. 2,4 and 7)	1 (1.22 ± 1.43)	1 (1.44 ± 1.46)	0.0038
IPSS voiding score (Nos. 3, 5 and 6)	1 (1.16 ± 1.58)	1 (1.33 ± 1.65)	0.1500
OABSS incontinence (No. 4)	1 (1.41 ± 1.05)	1 (1.43 ± 0.88)	0.2374

## 4 DISCUSSION

In this study, we compared ED patients categorized by severity and found that as ED symptoms worsened, both IPSS and OABSS total scores increased (*p* < 0.001) (Figures 1 and 2). Several mechanisms have been proposed to explain how LUTS patients develop ED.^7^ The first is a decrease in the production of nitric oxide (NO) and nitric oxide synthase (NOS). NO plays a crucial role in the relaxation of cavernous smooth muscle and blood vessels^7^ and is also believed to be involved in the contraction of the bladder neck and prostate smooth muscle during urination.^8^ The second factor is metabolic syndrome and autonomic nervous system hyperactivity. Metabolic syndrome is a risk factor for both ED and LUTS.^9^, ^10^ Additionally, metabolic syndrome, particularly hypertension, promotes sympathetic nervous system dominance.^11^ Sympathetic nervous system hyperactivity promotes contraction of the bladder neck and prostate smooth muscles and has been shown to induce ED and benign prostatic hyperplasia.^11^, ^12^, ^13^ The third mechanism is the activation of the Rho‐kinase pathway, which is involved in the contraction of prostate smooth muscle and penile corpus cavernosum smooth muscle^14^, ^15^, ^16^ The fourth factor is atherosclerosis of the pelvic vessels. It has been hypothesized that ischemic changes in the pelvic region lead to fibrosis, atrophy and reduced compliance of the bladder, prostate smooth muscle and penile corpus cavernosum smooth muscle.^17^, ^18^

In clinical studies, it has been reported in previous research that the presence of ED exacerbates LUTS. Blanker et al.^19^ evaluated the association between ED and LUTS in 1688 individuals in the Netherlands. The results, based on a multiple logistic regression analysis, indicated that ED was correlated with the severity of LUTS (mild LUTS vs. no symptoms: HR 1.8, 95% CI [0.8–4.3]; moderate LUTS vs. no symptoms: HR 3.4, 95% CI [1.4–8.4]; severe LUTS vs. no symptoms: HR 7.5, 95% CI [2.5–22.5]). Additionally, Choi et al.^6^ reported an association between ED and LUTS in a study of 1467 men aged 20–60 years in Korea, using the IIEF‐5 and IPSS. The results demonstrated that IIEF‐5 and IPSS scores were significantly negatively correlated. (*r* = −0.243, *p* < 0.001).

Regarding urinary symptoms and incontinence, the total IPSS score was higher in the group with more severe ED (*p* < 0.001). Regarding post‐void symptoms and urinary retention symptoms, no significant difference in the total IPSS score was observed between the mild ED group and the mild‐to‐moderate ED group. However, in the severe ED group (IIEF < 8), all items (post‐void symptoms, urinary retention symptoms, urinary symptoms and urinary incontinence) demonstrated higher scores in comparison to the other groups (*p* < 0.001). Several studies have reported an association between the severity of ED and LUTS. Elliot et al.^20^ evaluated 181 veterans in San Francisco using the IPSS and Sexual Health Inventory for Men (SHIM) and reported that only the total urinary symptom score was associated with ED (*p* = 0.001).

Terai et al.^21^ reported ED severity was associated only with urinary urgency (OR, 1.75) and nocturia (OR, 1.35) among the IPSS items. Additionally, in the aforementioned study by Choi et al.,^6^ the mild ED group reported higher scores than the normal group for all IPSS items, urinary symptoms, storage symptoms and IPSS total score. Furthermore, among the seven IPSS items, Item 5 (urinary force) demonstrated the strongest correlation with IIEF‐5 score (*r* = −0.243, *p* < 0.001). Previous reports have been inconsistent regarding which LUTS are associated with ED; however, this study found that severe ED worsens all LUTS. Additionally, urinary symptoms and incontinence worsened in proportion to the severity of ED.

In this study, evaluations were conducted separately for the younger age group (40s–50s) and older age group (over 70s). In the younger age group, the ED‐positive group (IIEF ≤ 21) demonstrated worsening of all LUTS compared with the ED‐negative group (IIEF ≥ 22) (*p* < 0.001). In contrast, among the elderly, only urinary retention symptoms demonstrated significant worsening between the ED‐positive and ED‐negative groups (*p* = 0.0038). Both ED and LUTS have been reported to be associated with metabolic syndromes, including hypertension, diabetes and hyperlipidemia, as risk factors.^6^, ^7^, ^9^, ^10^

But this study demonstrated that younger individuals with a lower metabolic syndrome risk had a stronger correlation between ED and LUTS in comparison to older individuals. This suggests that the severity of ED and LUTS is independently correlated with risk factors, such as age and metabolic syndrome.

Lastly, this cross‐sectional study included 4799 Japanese men. De Nunzio et al.^22^ conducted a systematic review of 46 studies on the relationship between ED and LUTS. The total number of cases across the 46 papers was 1017.5 (1720.55 ± 2246.85). Additionally, a study on the association between ED and IPSS in Asia was reported by Choi et al.^6^ in Korea, involving 1467 participants. Reports from Japan include studies by Nakamura et al.,^23^ involving 220 participants, and Terai et al.,^21^ involving 2084 participants. To the best of our knowledge, this study is the largest to date to report on the association between ED and LUTS in Asian populations. Additionally, the two aforementioned Japanese reports targeted individuals who visited medical institutions. In contrast, this study conducted a survey unrelated to whether individuals had visited medical institutions, making it a study that closely aligns with the general population.^21^, ^23^

This study had several limitations. First, as this study was based on a questionnaire survey, it was not possible to confirm the actual presence or absence of benign prostatic hyperplasia. Second, because this was a cross‐sectional study, it was not possible to determine which condition, ED or LUTS, developed first. Third, this study analyses whether ED and LUTS correlate without considering treatment history for either condition. However, this study analysed a large sample of 4799 individuals, and it is considered reasonable to evaluate the relationship between ED and LUTS in a cross‐sectional study.

## 5 CONCLUSION

The severity of ED was correlated with the severity of LUTS. Among the symptoms of LUTS, urinary symptoms and incontinence were found to correlate with the severity of ED and worsening of symptoms. In addition, severe ED worsened all symptoms of LUTS in comparison to no or mild‐to‐moderate ED.

## AUTHOR CONTRIBUTIONS

*Conceptualization*: Jun‐ichi Teranishi, Takashi Kawahara. Data collection: Akihito Hashizume, Daiki Ueno. *Investigation*: Hiroki Ito. *Methodology*: Hisashi Hasumi, Hiroji Uemura. *Visualisation*: Yasushi Yumura. *Validation*: Shinnosuke Kuroda. *Supervision*: Teppei Takeshima, Kazuhide Makiyama, Jun‐ichi Teranishi, Takashi Kawahara. All authors have read and agreed to the published version of the manuscript.

## CONFLICT OF INTEREST STATEMENT

The authors declare no conflicts of interest.

## Supporting information


**Table S1.** Medical history and social history of the study population.
