# Supplementary material for: Association of erectile dysfunction and male lower urinary tract symptoms in a Japanese cross‐sectional survey
Source: BJUI Compass. 2026 May 4;7(5):e70219. doi: 10.1002/bco2.70219 (PMC13138906; doi:10.1002/bco2.70219)
Supplement: Supplementary file 1 — Table S1. Medical history and social history of the study population. [file BCO2-7-e70219-s001.docx]

| sTable 1 Medical history and social history of the study population | | | | | |
| --- | --- | --- | --- | --- | --- |
|  | number(％) median(mean±SD) | | | |  |
|  | IIEF≧22 | IIEF-5 17-21 | IIEF-5 8-16 | IIEF-5 ＜８ | P value |
| Habitual smoking | 353(36.2%) | 279(33.3%) | 595(31.2%) | 244(22.6%) | *P*＜0.001 |
| Habitual drinking | 754(77.4%) | 621(74.2%) | 1378(72.1%) | 769(71.3%) | *P*＝0.007 |
| Medical history |  |  |  |  |  |
| Diabetes | 44(4.5％) | 62(7.4%) | 172(9.0%) | 174(16.1%) | *P*＜0.001 |
| Hypertention | 159(16.3%) | 172(20.5%) | 505(26.4%) | 391(36.3%) | *P*＜0.001 |
| Hyperlipidemia | 84(8.6%) | 84(10.0%) | 209(10.9%) | 160(14.8%) | *P*＜0.001 |
| Liver disease | 5(0.5%) | 14(1.7%) | 26(1.4%) | 18(1.4%) | *P*＝0.081 |
| Kidney disease | 5(0.5%) | 6(0.7%) | 36(1.9%) | 25(2.3%) | *P*＜0.001 |
| Heat disease | 11(1.1%) | 21(2.5%) | 75(3.9%) | 60(5.6%) | *P*＜0.001 |
| Mental illness | 25(2.6%) | 24(2.9%) | 76(4.0%) | 45(4.2%) | *P*＝0.104 |
